# Facilitated crystal handling using a simple device for evaporation reduction in microtiter plates

**DOI:** 10.1107/S1600576720016477

**Published:** 2021-02-01

**Authors:** Tatjana Barthel, Franziska U. Huschmann, Dirk Wallacher, Christian G. Feiler, Gerhard Klebe, Manfred S. Weiss, Jan Wollenhaupt

**Affiliations:** a Helmholtz-Zentrum Berlin, Macromolecular Crystallography, Albert-Einstein-Straße 15, 12489 Berlin, Germany; b Freie Universität Berlin, Institute for Chemistry and Biochemistry, Structural Biochemistry Group, Takustraße 5, 14195 Berlin, Germany; c Philipps-Universität Marburg, Institute of Pharmaceutical Chemistry, Drug Design Group, Marbacher Weg 6, 35032 Marburg, Germany; d Helmholtz-Zentrum Berlin, Department Sample Environment, Hahn-Meitner-Platz 1, 14109 Berlin, Germany

**Keywords:** evaporation reduction, crystal handling, crystal harvesting, crystallographic fragment screening

## Abstract

In the past two decades, most of the steps in a macromolecular crystallography experiment have undergone tremendous development with respect to speed, feasibility and increase of throughput. The part of the experimental workflow that is still a bottleneck, despite significant efforts, involves the manipulation and harvesting of the crystals for the diffraction experiment. Here, a novel low-cost device is presented that functions as a cover for 96-well crystallization plates. This device enables access to the individual experiments one at a time by its movable parts, while minimizing evaporation of all other experiments of the plate. In initial tests, drops of many typically used crystallization cocktails could be successfully protected for up to 6 h. Therefore, the manipulation and harvesting of crystals is straightforward for the experimenter, enabling significantly higher throughput. This is useful for many macromolecular crystallography experiments, especially multi-crystal screening campaigns.

## Abstract 2

A simple and low-cost device has been developed to minimize evaporation in microtiter plates for easy crystal handling and harvesting.

## 1. Introduction   

In the past two decades, X-ray crystallography, in particular macromolecular X-ray crystallography, has experienced an enormous boost with respect to automation and throughput. This is, in part, due to newer generation synchrotron facilities and increased sensitivity and fast readout of modern detectors (Leonarski *et al.*, 2018 ▸; Förster *et al.*, 2019 ▸; Owen *et al.*, 2016 ▸). Moreover, attempts to automate the entire process have led to advances in crystallization screening technologies, robot-assisted sample mounting at synchrotron beamlines, semi- or completely automated data collection, and more or less completely automated data processing and refinement procedures of the collected data (Shaw Stewart & Mueller-Dieckmann, 2014 ▸; Douangamath *et al.*, 2014 ▸; Bowler *et al.*, 2016 ▸; Powell, 2017 ▸). Efforts have also been directed towards the automation of crystal handling and crystal harvesting, tackling this bottleneck of high-throughput crystallography (Deller & Rupp, 2014 ▸; Cuttitta *et al.*, 2015 ▸; Wright *et al.*, 2021 ▸; Cipriani *et al.*, 2012 ▸). Most of these devices were built specifically for certain beamlines or laboratories, or have been commercialized, but at rather high costs due to the materials, mechanics and motors involved. Thus, they are only being used by a limited number of facilities and laboratories.

Without such devices at hand, crystal handling is still mainly manual work and time consuming. The crystals grown need to be individually harvested and flash-cooled in liquid nitro­gen before data collection. Prior to cooling, crystals are often further manipulated in the crystallization plate environment. Such manipulations, *i.e.* post-crystallization treatments where the crystals remain inside the drop, can involve heavy-metal derivatization for phasing experiments, dehydration to improve diffraction quality or soaking experiments with ligands (Heras & Martin, 2005 ▸; Rould, 2007 ▸). In particular for drug discovery projects the crystallographic screening of small-molecule compounds called fragments has recently been established as a high-throughput technique that requires the harvesting and preparation of a very large number of samples (Schiebel *et al.*, 2016 ▸; Krojer *et al.*, 2017 ▸; Lima *et al.*, 2020 ▸).

The entire process of harvesting and preparation of a crystalline sample for a diffraction experiment usually involves the cutting open of the foil that seals the crystallization plate to allow access to an individual well, the manipulation of crystals of that particular well according to the purpose of the experiment, and then the re-sealing of the respective well for incubation. At a later stage, the well is re-opened for harvesting of the samples. Taken together, this procedure is cumbersome, time consuming and limits the number of crystals that can be handled by the experimenter in a given time. Since most of the steps required for sample manipulation usually involve partial evaporation of liquids composing the crystallization drop, any attempt to optimize the process will have to account for evaporation. In this context, two recent developments need to be mentioned: The first is a plate lid with apertures (Zipper *et al.*, 2014 ▸). This was mainly developed for reducing evaporation during the drop-setting part of the experiment. The second development is the Crystal Shifter (Wright *et al.*, 2021 ▸), a motorized XY microscope stage developed to speed up crystal handling and simultaneously reduce evaporation.

Here we present a small and affordable device which reduces evaporation during crystal manipulation steps while at the same time allowing for easy crystal handling. It thus facilitates rapid crystal treatment and harvesting. It is applied as a temporary lid placed on top of a crystallization plate and can be used in combination with any typical laboratory microscope. It is currently customized for 3-lens 96-well MRC low-profile crystallization plates, but the design is easily adaptable and can be modified to fit any 96-well crystallization plate following the standard SBS footprint.

## 2. Design and assembly   

The design of the device is inspired by a sliding puzzle: *i.e.* individual vertically movable square tiles enable access to the individual wells on the crystallization plate. Concurrently, the tiles protect the other wells, which have to remain covered and thus protected from evaporation, ideally for several hours.

**Figure 1:**
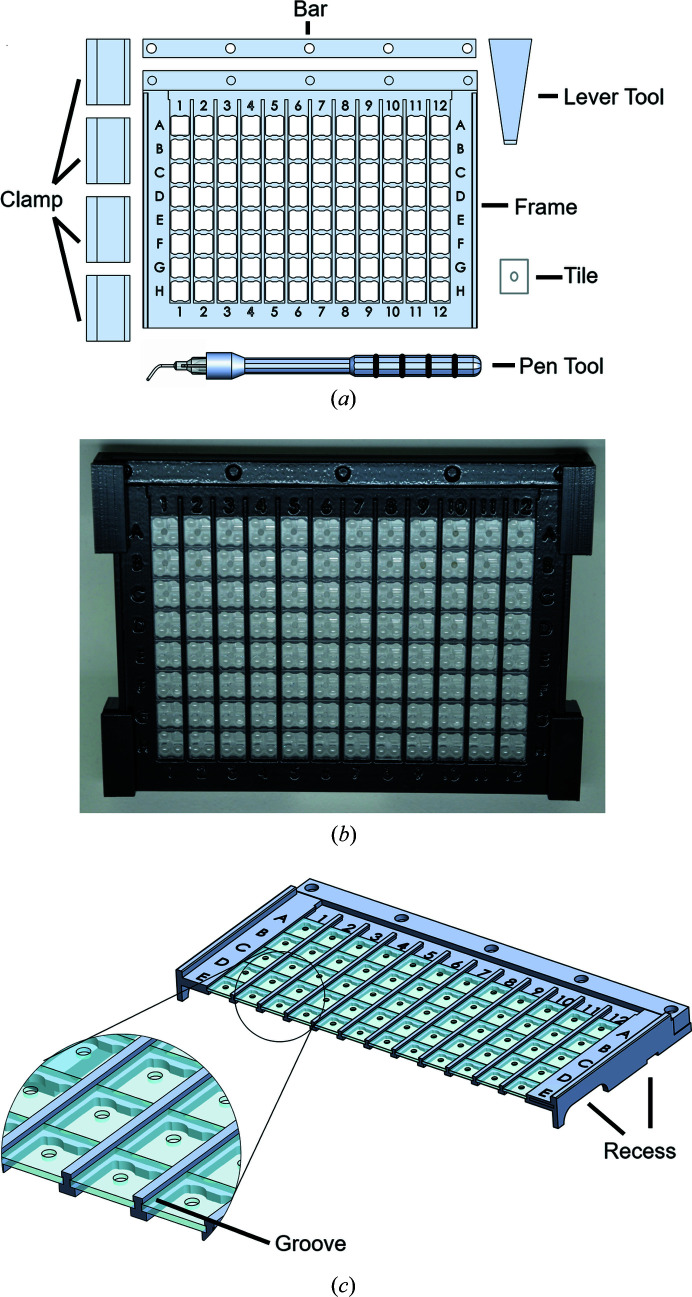
Parts and assembly of the device. (*a*) Schematic overview of all parts of the frame as well as an acrylic glass tile and the pen and lever tools. (*b*) Photograph of the fully assembled frame. It was placed on a 3-lens 96-well MRC low-profile crystallization plate and secured by four clamps. (*c*) The device, seen from another side, as a schematic cross section showing the recesses available on the side. The larger recess can be used via the thumb to hold the plate while lifting the frame; the smaller recesses fit the lever tool to loosen the frame before completely lifting it up. In the close-up view (circle) the groove with the acrylic glass tiles inserted is visible.

The device consists of a frame, a bar, sliding clamps, a lever tool made from 3D-printed plastic and 96 acrylic glass tiles (Fig. 1 ▸). The set is completed by a two-piece pen tool, the handle of which is 3D-printed as well. The top part is designed to accommodate a bent cannula with a rounded tip. All 3D-printed plastic parts are made from thermoplastic (VeroBlackPlus, stratasys) using an Objet30 Pro printer. The tiles are made from 0.8 mm-thick acrylic glass plates and excized using a Universal Laser Systems M20 laser. The laser is also used for engraving the tiles in the middle, *i.e.* adding little dips of 1.63 mm diameter. These serve as optional handles when the pen tool is used. After production and cleaning of the parts, the tiles are inserted into the grooves of the frame so that they can be moved vertically. The bar prevents the tiles from accidentally falling off or being slid off the frame. Thus, the tiles are kept inside the frame by the grooves and the bar. The tiles can be moved either with the tip of a finger or, alternatively, using the pen tool to avoid touching them by hand. The production of the frame, including the lasering of the acrylic glass tiles, the printing and cleaning of all 3D-printed parts, and the insertion of the 96 acrylic glass tiles into the frame, takes approximately 10 h per set.

The frame edges are labeled with the corresponding well numbers and letters, helping the experimenter to keep track of each position. In its current design, the frame only fits onto 3-lens 96-well MRC low-profile crystallization plates. The frame is placed onto a prepared crystallization plate after removal of the respective sealing foil. It can be fixed onto the plate without the need for adhesives or grease using the provided clamps. For the safe removal of the frame from the plate, recesses are available on the left and right sides. The large recesses are provided to secure the plate with the thumb. The small recesses can be used in combination with the lever tool, which enables the frame to be lifted off the crystallization plate by a simple turning movement. The removal of the frame from the plate is not adversely affected by any potentially remaining minute amounts of adhesive from the crystallization foil removed earlier. The entire handling process of setting up the frame on the crystallization plate, possible ways of tile movement and removal of the frame from the crystallization plate are visualized in Video S1 of the supporting information.^1^

## 3. Application   

**Figure 2:**
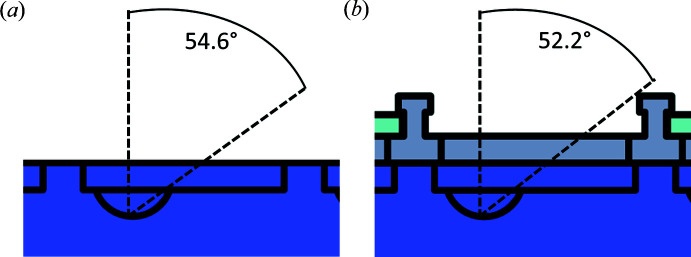
Schematic view of usable space with the device. A side view of one well of a 3-lens 96-well MRC low-profile crystallization plate (purple) is shown without or with the frame (gray). The half circle inside the plate represents one of the lenses of the crystallization plate. Usually the protein crystals will be located towards the bottom of the lens, and thus the angle is measured from this point. As the user will usually choose to work from the side which is most comfortable and provides the largest space for manipulating and harvesting, the angles from the right side are compared. (*a*) Plate without the frame. The angle shown depicts the possible working angle without the frame of 54.6°. (*b*) With the frame (gray) placed on top of a crystallization plate, the angle slightly decreases to 52.2°. Using the frame, a reduction of only 2.4° in angular movement range has to be accepted by the experimenter.

By placing the frame on top of the crystallization plate, each well is sealed individually, and the wells can be accessed one at a time. The transparent acrylic glass tiles allow the observation of each experiment in its sealed compartment. The engraved depressions on the acrylic glass tiles do not impair the view of the drops. Due to the height of the frame, the working angle at which it is possible to reach a crystallization drop inside a 3-lens 96-well MRC low-profile crystallization plate decreases by less than three degrees (Fig. 2 ▸). Such angles are still comfortable for handling/harvesting crystals under a typical laboratory microscope. The angle was estimated by considering the usual position of protein crystals on the crystallization plate lens. It was assumed that manipulation and harvesting would be performed from the right side, as this gives the maximum space possible with and without the assembled device. If it is difficult to manipulate or harvest a protein crystal from this specific side, it is of course possible to move/rotate the assembly of the plate with the frame to a position that allows for easy handling.

## 4. Evaporation reduction   

The aim of this device is to work with most solutions, except those containing a volatile material as the main component. Reliable evaporation reduction can be achieved for 1 to 6 h, *i.e.* a typical working session, after the evaporation equilibrium has been reached. If an experiment requires longer incubation times, the frame should be removed and the crystallization plate re-sealed with a standard foil used in macromolecular crystallography. In order to estimate the performance of the device, evaporation experiments were conducted. The working time was assessed while experiments were protected by the acrylic glass tiles. The frame was tested by observing the evaporation of different solutions. Soaking solutions from already performed crystallographic fragment screening (CFS) campaigns and solutions from a typical crystallization screen consisting of 96 different crystallization solutions (JSCG Core Suite II, Quiagen) were tested to obtain an overview of the usability range.

### 4.1. Evaporation reduction – CFS soaking solutions   

In the case of the experiments concerning the evaporation of soaking solutions a 3-lens 96-well MRC low-profile crystallization plate was used with a 40 µl reservoir and 0.4 µl drops in the tested wells. The reservoir was pipetted before the plate was covered by the frame. The drops were set manually after covering the plate with the frame and accessing the individual wells by appropriate tile movements. Each solution was tested in three individual experiments while care was taken to distribute the used wells across the plate (corner versus edge versus center). Subsequently, the drops were observed visually over time at a fixed magnification and focus. The plate was placed on a motorized XY table underneath the microscope lens, allowing plate movements under the microscope in a defined and reproduceable manner. As a metric for the extent of evaporation, the diameter of the drops was used. Photographs were taken through the transparent tiles while the experiment was sealed and left undisturbed over the entire course.

**Figure 3:**
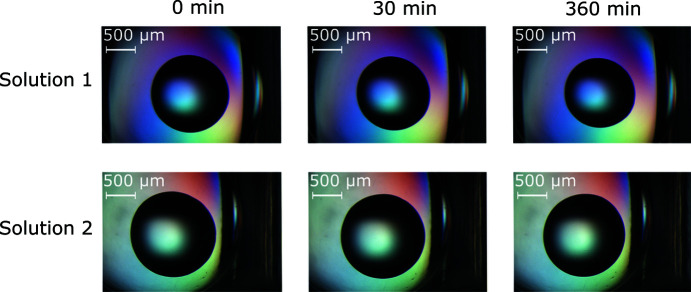
Evaporation test of the device versus exemplary CFS soaking solutions. Depicted are two different soaking solutions used in CFS campaigns at BESSY II, HZB. Both solutions (Solution 1 and Solution 2) already contain DMSO and a cryoprotectant. Immediately after placing the frame on top of the plate and finishing setting all drops, an image at 0 min was taken of each drop as a starting point. The evaporation observations were performed over 6 h at several intermediate time points. The images were taken with the same microscope and settings each time. Depicted here are the 30 and 360 min time points for both conditions tested. Little to no change is observed even after 360 min, thus showing high evaporation protection of the solutions by the frame.

The CFS soaking solutions usually contain the same components of the crystallization conditions and, in addition, a certain amount of dimethyl sulfoxide (DMSO) and cryoprotectant. In a CFS campaign, either crystals are transferred into the soaking solution or DMSO and cryoprotectants are added to the crystallization drop. The crystals will usually be soaked for 30 min to about 24 h, depending on the circumstances. For our purposes, the crystals needed to be transferred into a soaking drop and soaked for about 16 h overnight. The overnight soaking was performed in a foil-sealed plate. Handling or harvesting of 96 crystals usually takes between 1 and 2 h depending on the experimenters’ experience and the quality/robustness of the crystals. This means the solutions should optimally stay almost unchanged within that time window to allow for transfer and harvesting of 96 crystals in one session using the frame. Two CFS soaking solutions from previous campaigns (Wollenhaupt *et al.*, 2020 ▸) were tested as examples (Fig. 3 ▸). Solution 1 contains 17%(*w*/*v*) PEG 4000, 180 m*M* Tris pH 8.5, 180 m*M* Li_2_SO_4_, 5%(*v*/*v*) 1,2-propane­diol and 8%(*v*/*v*) DMSO; and solution 2 contains 19.8%(*w*/*v*) PEG 4000, 68 m*M* sodium acetate pH 4.6, 68 m*M* ammonium acetate, 19.3%(*v*/*v*) glycerol and 9%(*v*/*v*) DMSO. The drops show minimal evaporation over the course of 6 h, which is a reasonable time frame to carry out crystal soaking and harvesting of about 300 crystals. The results were independent of the position of the wells used on the plate. Several CFS campaigns at BESSY II, Helmholtz-Zentrum Berlin (HZB), have already been successfully performed using the device. Users typically reported comfortable usage and significant speed-up of crystal handling.

### 4.2. Evaporation reduction – 96-well crystallization solution screen   

In protein crystallization experiments, a plethora of crystallization conditions are screened in order to derive diffracting protein crystals. Therefore, numerous crystallization screens have been assembled by combining solutions that potentially facilitate crystal formation (Jancarik & Kim, 1991 ▸). The individual solutions typically consist of a combination of precipitant and buffer, sometimes with salts and additives. Considering all possible combinations, an endless number of possible crystallization conditions can be created. Therefore, it is not feasible to perform a comprehensive, all-encompassing experimental evaporation test of all possible combinations. For a first overview, we tested a typical commercial 96-well screen, the JSCG Core Suite II (Quiagen), which includes many common classes of solutions used in macromolecular crystallography (Table S1 of the supporting information). A 3-lens 96-well MRC low-profile crystallization plate was used with 40 µl reservoir and 0.4 µl drops. The reservoir and the sitting drops were pipetted using a 96-syringe head pipetting robot (Gryphon, Art Robbins Instruments) (*i.e.* the drops were pipetted in parallel) before the plate was covered with the frame. The drop size was recorded as described in Section 4.1, leaving the experiments closed.

**Figure 4:**
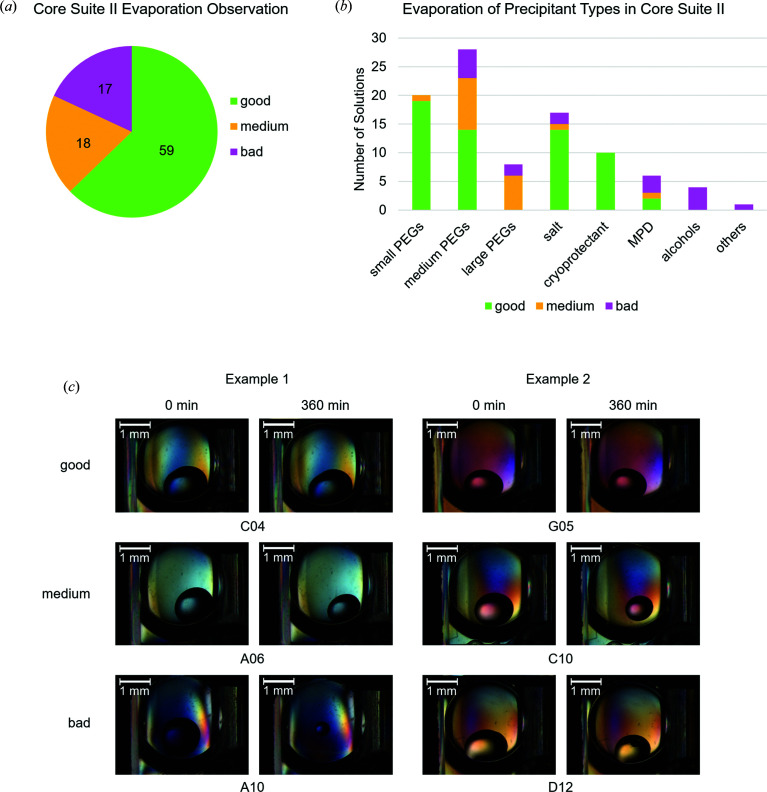
Evaporation test of the device versus the JSCG Core Suite II. The evaporation observations were performed for 6 h following the method described in Fig. 3 ▸. (*a*) Pie chart illustrating the number of solutions categorized as good (green), medium (yellow) and bad (purple). (*b*) 94 solutions (excluding B08 and B10 due to their low surface tension) were reduced to nine groups that represent different types of precipitants. They show slight tendencies for certain precipitant types. (*c*) Two examples are shown for each category, each with the 0 and 360 min time points. These time points were used to assess the overall reduction of drop size or, in the case of D12, to detect the appearance of phase separation.

A typical observation during the first 30 min was that the freshly set drop of each solution decreased slightly in size, probably owing to reaching the evaporation equilibrium inside the sitting-drop experiment. The final decrease of the drop diameter after 360 min was used to assess evaporation. Usually, the drops are not perfectly circular from the top view, but often elliptic. Therefore, the mean between the major and the minor axes of the observed ellipse was taken. Three independent repetitions of the experiment were performed, and the relative drop diameter reductions of those experiments were averaged for each respective solution. Observations of changes in drop sizes were classified into three groups: ‘good’ – a reduction of less than 15% of the drop diameter; ‘medium’ – a reduction of about 15–30% of the drop diameter; and ‘bad’ – a reduction of more than 30% of the drop diameter or drops that show phase separation or crystallization of individual solution components (Fig. 4 ▸). In two cases, the conditions B08 and B10 (Table S1), no useful diameter could be measured due to the small surface tension of the solution, which created a large flat drop. These drops were therefore not included in the analysis. From the 94 solutions of the screen used for the analysis, 59 of the solutions were found to be good, 18 were medium and 17 were bad (Table S1). This shows that the device works well for many solutions of this screen. In the experiment, certain tendencies could be seen for the precipitant compound. With a focus on PEGs, which are rather abundant in the screen, it was observed that solutions including smaller PEGs (PEG 200 to PEG 600) are more often categorized as good than larger PEGs (PEG 8000 to PEG 20000). In the case of PEG 6000, additional salt can help to keep the drop size stable. For PEG 1000 to PEG 3350 salt addition seems to have no apparent effect. A salt abundantly used as a precipitant is ammonium sulfate. The categorization is usually good as long as the concentration is above 0.4 *M*. There is only one exception, where 0.8 *M* ammonium sulfate is mixed with 0.1 *M* citric acid pH 4.0 (category medium). Typical alcohols in the screen are ethanol and iso-propanol and solutions containing these alcohols as their main component fell into the bad category. Typical cryoprotectants used in the screen solutions are glycerol, ethyl­ene glycol and 1,2-propane­diol. Those conditions were categorized as good. The evaporation results of some precipitants cannot be this easily generalized. In the case of PEG 4000, the solution was usually mixed with glycerol, which might be the actual ingredient turning these solutions into good ones. A similar consideration can be made for 2-methyl-2,4-pentanediol (MPD), where the glycerol-containing solutions were classified as good and the MPD solutions without glycerol as bad.

## 5. Discussion   

All in all, the experiments show that in most cases of the JSCG Core Suite II solutions the user can reliably work for at least 1 h, which is often sufficient to carry out manipulation and harvesting of up to 100 crystals. However, the observations also indicate how unpredictable the evaporation properties seem to be, judging from their components alone. We tried to name certain trends but given the relatively small variety of solutions tested (compared with the vast number of possible combinations in crystallography) no general trends can be applied. Intuitively, the evaporation properties should be connected to the vapor pressure and relative humidity of the individual solutions. There have been several approaches to predict the relative humidity of crystallization conditions on the basis of their main component, to perform controlled dehydration experiments of single crystals outside the crystallization drop (Wheeler *et al.*, 2012 ▸; Bowler *et al.*, 2015 ▸, 2017 ▸).

Online applets for convenient use of the calculations described in the above publications are publicly available (http://go.esrf.eu/UsersAndScience/Experiments/MX/How_to_use_our_beamlines/forms). However, relative humidity values predicted with these formulas based on the main components of the JSCG Core II Suite solutions did not show an obvious relationship by casual comparison with our experimental results of evaporation in crystallization drops. Therefore, factors other than the relative humidity are likely to additionally influence the evaporation properties of crystallization drops in microtiter plate environments. Nevertheless, the device demonstrated excellent performance for more than 50% of the 96 crystallization solutions and the tested soaking solutions from different CFS campaigns. Thus, it could be argued that, in most cases, the device is likely to perform well. However, it is preferable that each solution intended for use for extended time periods should be tested beforehand. This is especially worthwhile for experiments with enhanced throughput like CFS where the composition of the soaking solution is invariant by design.

Unlike the other approach to evaporation reduction specifically made for the setup of crystallization plates mentioned earlier (Zipper *et al.*, 2014 ▸), the frame provides sufficient angular space to conveniently manipulate crystals inside the sitting drops. Furthermore, it enables the user to leave open only the well which is being worked on. All other wells remain covered during the entire experiment. In contrast to other devices specifically designed to increase speed for crystal harvesting, like the semi-automated Crystal Shifter (Wright *et al.*, 2021 ▸), the frame does not come with integrated automation or digital sample tracking. Still, it has several other advantages. Firstly, it has a much lower production cost as it is simple, made of plastic/acrylic material, and lacks any metal parts, electronics or motors. Secondly, the frame is small and light; thus, it can be transported easily. Although the Crystal Shifter remains the best solution in terms of speed, the frame is more versatile and more widely applicable.

## 6. Summary   

A novel evaporation-protecting device, which we conveniently term the EasyAccess Frame, which minimizes evaporation to facilitate crystal handling and harvesting, is presented. It is small and light and therefore well suited for transportation. The current version of the EasyAccess Frame is designed to fit the 3-lens 96-well MRC low-profile crystallization plate; however, the design can be adapted to any other 96-well crystallization plate in SBS format in the future.

Widely applied experiments in macromolecular crystallography are, for instance, heavy-atom derivatization for phasing and in-drop dehydration experiments to improve diffraction properties of crystals. All these applications can be conveniently performed using the novel tool presented here. It is also possible to customize the frame to a certain extent for different applications. For example, in the case of photosensitive protein crystals, the transparent colorless acrylic glass tiles could be exchanged for colored ones, protecting the crystals from a particular wavelength of light, while still allowing the user to observe the enclosed experiment. For left-handed users, a frame could be designed that fits onto the plate rotated 180° horizontally. All in all, the EasyAccess Frame reduces evaporation in microtiter plates while easing the access to the individual experiments on the plates. In this way it speeds up manual manipulation and harvesting of protein crystals, thereby benefitting X-ray crystallography experiments in general, but especially enhanced-throughput screening experiments. It does not need any special equipment, is re-usable for many different experiments and thus is ideal for every laboratory.

## 7. Disclosures   

A patent application regarding the reported frame device has been filed by Helmholtz–Zentrum Berlin with the German Patent and Trademark Office (registration No. DE 10 2018 111 478.8). Additionally, an international patent application via the PCT route, using the priority of the German patent, has been filed. Furthermore, the handle of the pen tool is protected by a registered design (registration No. DE 40 2018 100 962-0001).

## Supplementary Material

Setting up of the device on the plate, moving of the tiles and removal of the frame from the plate. DOI: 10.1107/S1600576720016477/gj5257sup1.mp4

The JSCG Core Suite II crystallization cocktails listed with their individual components per well position, the corresponding precipitant category, the measured average reduction of the drop diameter, a comment if phase separation occurred and the classification. DOI: 10.1107/S1600576720016477/gj5257sup2.pdf

## Footnotes

1 Before placing the frame on top of the crystallization plate certain steps need to be performed that are not included in the video. These steps depend on the individual experiment. In the case where foil seals the plate, the foil needs to be removed beforehand. After removing the frame from the plate, the latter can be sealed again by a foil, *e.g.* for further incubation steps, or the plate can be discarded.
