# Supplementary material for: Facilitated crystal handling using a simple device for evaporation reduction in microtiter plates
Source: J Appl Crystallogr. 2021 Feb 1;54(Pt 1):376–82. doi: 10.1107/S1600576720016477 (PMC7941301; doi:10.1107/S1600576720016477)
Supplement: Supplementary file 2 [file j-54-00376-sup2.pdf]

JOURNAL OF
APPLIED
CRYSTALLOGRAPHY

Volume 54 (2021)

Supporting information for article:

Facilitated crystal handling using a simple device for evaporation reduction in microtiter plates

Tatjana Barthel, Franziska U. Huschmann, Dirk Wallacher, Christian G. Feiler, Gerhard Klebe, Manfred S. Weiss and Jan Wollenhaupt

Well	Main component	Component 2	Component 3	Buffer	Precipitent category	Mean diameter reduction (n=3) [%]	Data range (n=3) [%]	Comment	Category
**A01**	20%(w/v) PEG 8000	0.2 M Sodium chloride		0.1 M CAPS pH 10.5	large PEG	18	16 - 19		medium
**A02**	1.26 M Ammonium sulfate	0.2 M Sodium chloride		0.1 M CHES pH 9.5	salt	12	10 - 14		good
**A03**	1.0 M Sodium citrate			0.1 M CHES pH 9.5	salt	1	-2 - 5		good
**A04**	10%(w/v) PEG 8000	0.2 M Sodium chloride		0.1 M CHES pH 9.5	large PEG	23	15 - 30		medium
**A05**	10%(w/v) PEG 20000	2%(v/v) 1,4-Dioxane		0.1 M Bicine pH 9.0	large PEG	28	26 - 29		medium
**A06**	20%(w/v) PEG 550 MME	0.1 M Sodium chloride		0.1 M Bicine pH 9.0	small PEG	16	14 - 18		medium
**A07**	10%(w/v) PEG 6000	1.0 M Lithium chloride		0.1 M Bicine pH 9.0	medium PEG	15	13 - 17		medium
**A08**	20%(v/v) PEG 300	5%(w/v) PEG 8000	10%(v/v) Glycerol	0.1M Tris pH 8.5	small PEG	5	3 - 7		good
**A09**	20%(w/v) PEG 2000 MME	0.01 M Nickel chloride		0.1 M Tris pH 8.5	medium PEG	10	9 - 10		good
**A10**	20%(v/v) Ethanol			0.1 M Tris pH 8.5	alcohol	63	61 - 64		bad
**A11**	2.0 M Ammonium dihydrogen phosphate			0.1 M Tris-HCl pH 8.5	salt	11	9 - 14		good
**A12**	8%(w/v) PEG 8000			0.1 M Tris-HCl pH 8.5	large PEG	13	12 - 15	phase separation	bad
**B01**	2.0 M Ammonium sulfate			0.1 M Tris-HCl pH 8.5	salt	9	1 - 14		good
**B02**	40%(v/v) PEG 400	0.2 M Lithium sulfate		0.1M Tris pH 8.5	small PEG	4	2 - 7		good
**B03**	10%(w/v) PEG 8000	0.2 M Calcium acetate		0.1 M Imidazole pH 8.0	large PEG	27	23 - 33		medium
**B04**	35%(v/v) MPD	0.2 M Magnesium chloride		0.1 M Imidazole pH 8.0	MPD	13	6 - 19		medium
**B05**	20%(w/v) PEG 6000	1.0 M Lithium chloride		0.1 M Tris pH 8.5	medium PEG	8	8 - 10		good
**B06**	20%(w/v) PEG 6000			0.1 M Tris pH 8.5	medium PEG	20	19 - 22		medium
**B07**	20%(w/v) PEG 3350	0.2 M Lithium Acetate			medium PEG	18	14 - 20		medium
**B08**	40%(v/v) MPD	0.2 M Magnesium chloride		0.1M Imidazole pH 8.0	MPD	-	-	not possible to measure	-
**B09**	15%(v/v) Ethanol	0.2 M Magnesium chloride		0.1 M HEPES pH 7.5	alcohol	36	34 - 38		bad
**B10**	70%(v/v) MPD			0.1 M HEPES pH 7.5	MPD	-	-	not possible to measure	-
**B11**	17%(w/v) PEG 4000	15%(v/v) Glycerol	8.5%(v/v) Isopropanol	0.085 M Sodium HEPES pH 7.5	medium PEG	5	3 - 7		good
**B12**	25%(v/v) Glycerol	0.6 M sodium dihydrogen phosphate/0.6 M potassium dihydrogen phosphate		0.075 M Sodium HEPES pH 7.5	cryoprotectant	2	1 - 3		good
**C01**	27%(v/v) PEG 400	10%(v/v) Glycerol	0.18 M Magnesium chloride	0.09 M Sodium HEPES pH 7.5	small PEG	4	1 - 7		good
**C02**	2.0 M Ammonium sulfate	2%(v/v) PEG 400		0.1 M Sodium HEPES pH 7.5	salt	5	4 - 7		good
**C03**	30%(v/v) PEG 400	0.2 M Magnesium chloride		0.1 M Sodium HEPES pH 7.5	small PEG	5	2 - 9		good
**C04**	50%(v/v) PEG 200	0.2 M Sodium chloride		0.1M Na/K phosphate pH 6.2	small PEG	0	0 - 1		good
**C05**	20%(w/v) PEG 3350	0.2 M Sodium fluoride			medium PEG	16	15 - 18		medium
**C06**	2.0 M Ammonium sulfate	0.2 M Lithium sulfate		0.1 M Tris pH 7.0	salt	1	0 - 2		good
**C07**	40%(v/v) PEG 300	0.2 M Calcium acetate		0.1M Sodium cacodylate pH 6.5	small PEG	1	-1 - 2		good
**C08**	20%(w/v) PEG 1000			0.1 M Tris pH 7.0	medium PEG	16	12 - 18		medium
**C09**	10%(w/v) PEG 6000	1.0 M Lithium chloride		0.1 M HEPES pH 7.0	medium PEG	12	11 - 13		good
**C10**	10%(w/v) PEG 6000			0.1 M HEPES pH 6.5	medium PEG	26	25 - 27		medium
**C11**	40%(v/v) PEG 400	0.2 M Sodium chloride		0.1M Na/K phosphate pH 6.2	small PEG	1	1 - 1		good
**C12**	50%(v/v) PEG 200			0.1M Sodium citrate pH 5.5	small PEG	1	1 - 1		good
**D01**	25%(v/v) 1,2-Propanediol	10%(v/v) Glycerol		0.1M Na/K phosphate pH 6.2	cryoprotectant	4	3 - 6		good
**D02**	20%(w/v) PEG 3350	0.2 M Sodium nitrate			medium PEG	19	16 - 22		medium
**D03**	50%(v/v) PEG 200	0.05 M Lithium sulfate		0.1M Tris pH 7.0	small PEG	1	0 - 1		good
**D04**	20%(w/v) PEG 3350	0.2 M Potassium sulfate			medium PEG	23	21 - 27	crystallization	bad
**D05**	0.2 M Magnesium formate				salt	48	46 - 51		bad
**D06**	40%(v/v) PEG 600			0.1MSodium citrate pH 5.5	small PEG	3	2 - 3		good
**D07**	20%(w/v) PEG 1000	0.2 M Magnesium chloride		0.1 M Sodium cacodylate pH 6.5	medium PEG	13	11 - 14		good
**D08**	10%(w/v) PEG 3000	0.2 M Magnesium chloride		0.1 M Sodium cacodylate pH 6.5	medium PEG	23	19 - 27		medium
**D09**	30%(v/v) PEG 400	0.2 M Lithium sulfate		0.1 M Sodium cacodylate pH 6.5	small PEG	3	2 - 5		good
**D10**	2.0 M Ammonium sulfate	0.2 M Sodium chloride		0.1 M Sodium cacodylate pH 6.5	salt	1	1 - 1		good
**D11**	12%(w/v) PEG 20000			0.1 M MES pH 6.5	large PEG	28	28 - 29		medium
**D12**	20%(w/v) PEG 3350	0.2 M Lithium sulfate			medium PEG	11	11 - 12	phase separation	bad

Well	Main component	Component 2	Component 3	Buffer	Precipitent category	Mean diameter reduction (n=3) [%]	Data range (n=3) [%]	Comment	Category
**E01**	20%(w/v) PEG 1000	0.2 M Sodium chloride		0.1 M Na/K phosphate pH 6.2	medium PEG	19	16 - 24	phase separation	bad
**E02**	10%(v/v) MPD			0.1 M MES pH 5.0	MPD	41	40 - 42		bad
**E03**	20%(w/v) PEG 6000	1.0 M Lithium chloride		0.1 M MES pH 6.0	medium PEG	8	5 - 11		good
**E04**	10%(w/v) PEG 6000	1.0 M Lithium chloride		0.1 M MES pH 6.0	medium PEG	10	8 - 11		good
**E05**	5%(w/v) PEG 6000			0.1 M MES pH 5.0	medium PEG	42	41 - 44		bad
**E06**	25%(v/v) 1,2-Propanediol	10%(v/v) Glycerol	0.2 M Zinc acetate	0.1M Imidazole pH 8.0	cryoprotectant	3	2 - 4		good
**E07**	40%(v/v) PEG 600	0.2 M Zinc acetate		0.1M Imidazole pH 8.0	small PEG	3	2 - 4		good
**E08**	30%(v/v) PEG 600	10%(v/v) Glycerol	0.5 M Ammonium sulfate	0.1M Tris pH 7.0	small PEG	1	1 - 1		good
**E09**	1.0 M Lithium sulfate	0.5 M Ammonium sulfate		0.1 M Sodium citrate pH 5.6	salt	13	12 - 13		good
**E10**	30%(w/v) PEG 4000	0.2 M Ammonium acetate		0.1 M Sodium citrate pH 5.6	medium PEG	6	4 - 8	phase separation	bad
**E11**	24%(w/v) PEG 1500	20%(v/v) Glycerol			medium PEG	2	1 - 3		good
**E12**	40%(v/v) PEG 300	0.2 M Sodium chloride		0.1M Sodium acetate pH 4.5	small PEG	2	2 - 3		good
**F01**	35%(v/v) MPD	10%(v/v) Glycerol		0.1M Sodium acetate pH 4.5	MPD	9	7 - 10		good
**F02**	40%(v/v) PEG 300			0.1M Phosphate-citrate pH 4.2	small PEG	3	2 - 4		good
**F03**	50%(v/v) Ethylene glycol	5%(w/v) PEG 1000		0.1M Sodium acetate pH 4.5	cryoprotectant	1	0 - 2		good
**F04**	30%(v/v) PEG 200	0.1 M Sodium chloride		0.1M Sodium acetate pH 4.5	small PEG	5	3 - 8		good
**F05**	40%(v/v) 1,2-Propanediol			0.1M Sodium acetate pH 4.5	cryoprotectant	3	3 - 4		good
**F06**	40%(v/v) Ethylene glycol			0.1M Sodium acetate pH 4.5	cryoprotectant	2	1 - 2		good
**F07**	10%(v/v) MPD			0.1 M Sodium acetate pH 5.0	MPD	31	27 - 33		bad
**F08**	2.4 M Ammonium sulfate			0.1 M Citric acid pH 4.0	salt	4	3 - 5		good
**F09**	1.6 M Ammonium sulfate			0.1 M Citric acid pH 4.0	salt	9	8 - 9		good
**F10**	0.8 M Ammonium sulfate			0.1 M Citric acid pH 4.0	salt	23	17 - 33		medium
**F11**	20%(w/v) PEG 6000	1.0 M Lithium chloride		0.1 M Citric acid pH 5.0	medium PEG	7	6 - 8		good
**F12**	25%(v/v) 1,2-Propanediol	5%(w/v) PEG 3000	10%(v/v) Glycerol	0.1M Phosphate-citrate pH 4.2	cryoprotectant	1	1 - 1		good
**G01**	2.0 M Ammonium sulfate	5%(v/v) Isopropanol			salt	12	8 - 16		good
**G02**	2.0 M Ammonium sulfate				salt	10	9 - 13		good
**G03**	40%(v/v) PEG 400	0.2 M Magnesium chloride		0.1M MES pH 5.5	small PEG	3	1 - 5		good
**G04**	1.0 M Hexanediol	0.01 M Cobalt chloride		0.1 M Sodium acetate pH 4.6	alcohol	36	33 - 38		bad
**G05**	1.6 M Ammonium sulfate	20%(v/v) Glycerol		0.08 M Sodium acetate pH 4.6	salt	1	0 - 1		good
**G06**	30%(v/v) Glycerol	5.6%(w/v) PEG 4000		0.07 M Sodium acetate pH 4.6	cryoprotectant	4	2 - 8		good
**G07**	30%(v/v) Glycerol	14%(v/v) Isopropanol	0.14 M Calcium chloride	0.07 M Sodium acetate pH 4.6	cryoprotectant	7	5 - 8		good
**G08**	20%(w/v) PEG 4000	20%(v/v) Glycerol	0.16 M Ammonium sulfate	0.08 M Sodium acetate pH 4.6	medium PEG	2	1 - 3		good
**G09**	27%(v/v) MPD	10%(v/v) Glycerol	0.018 M Calcium chloride	0.09 M Sodium acetate pH 4.6	MPD	7	4 - 9		good
**G10**	2.0 M Ammonium sulfate			0.1 M Sodium acetate pH 4.6	salt	8	7 - 9		good
**G11**	10%(w/v) PEG 3000	0.2 M Zinc acetate		0.1 M Sodium acetate pH 4.5	medium PEG	29	25 - 34		medium
**G12**	20%(v/v) PEG 300	10% Glycerol	0.2 M Ammonium sulfate	0.1M Phosphate-citrate pH 4.2	small PEG	3	2 - 5		good
**H01**	30%(v/v) PEG 400	0.2 M Calcium acetate		0.1 M Sodium acetate pH 4.5	small PEG	9	6 - 11		good
**H02**	30%(w/v) PEG 8000	0.2 M Lithium sulfate		0.1 M Sodium acetate pH 4.5	large PEG	11	5 - 17	phase separation	bad
**H03**	25%(v/v) Ethylene glycol				cryoprotectant	15	13 - 17		good
**H04**	10%(v/v) Isopropanol	0.2 M Lithium sulfate		0.1 M Phosphate-citrate pH 4.2	alcohol	40	35 - 44		bad
**H05**	20%(w/v) PEG 8000	0.2 M Sodium chloride		0.1 M Phosphate-citrate pH 4.2	large PEG	15	12 - 17		medium
**H06**	10%(w/v) PEG 1000	10%(w/v) PEG 8000			medium PEG	14	11 - 16		good
**H07**	25.5%(w/v) PEG 4000	15%(v/v) Glycerol	0.17 M Ammonium sulfate		medium PEG	2	2 - 2		good
**H08**	30%(w/v) PEG 1500				medium PEG	8	6 - 10		good
**H09**	0.4 M Ammonium dihydrogen phosphate				salt	45	44 - 47		bad
**H10**	35%(v/v) 1,4-Dioxane				others	81	78 - 87		bad
**H11**	10%(v/v) MPD			0.1 M Citric acid pH 2.5	MPD	58	28 - 74		bad
**H12**	20%(w/v) PEG 6000			0.1 M Citric acid pH 2.5	medium PEG	11	8 - 16		good
